# The validation study on a three-dimensional burn estimation smart-phone application: accurate, free and fast?

**DOI:** 10.1186/s41038-018-0109-0

**Published:** 2018-02-27

**Authors:** A. K. W. Cheah, T. Kangkorn, E. H. Tan, M. L. Loo, S. J. Chong

**Affiliations:** 1 Department of Plastic, Reconstructive and Aesthetic Surgery, Singapore General Hospital, 1 Outram Road, Bukit Merah, Singapore; 2 Department of Plastic and Reconstructive Surgery, Chonburi Hospital, 69 Sukhumvit Road, Muang Chonburi, Thailand; 3 Navy Medical Service, Republic of Singapore Navy, 210 Tanah Merah Coast Road, Singapore, Singapore

**Keywords:** Burns, Estimation, Total body surface area burned, 3D Burn Resuscitation application

## Abstract

**Background:**

Accurate total body surface area burned (TBSAB) estimation is a crucial aspect of early burn management. It helps guide resuscitation and is essential in the calculation of fluid requirements. Conventional methods of estimation can often lead to large discrepancies in burn percentage estimation. We aim to compare a new method of TBSAB estimation using a three-dimensional smart-phone application named 3D Burn Resuscitation (3D Burn) against conventional methods of estimation—Rule of Palm, Rule of Nines and the Lund and Browder chart.

**Methods:**

Three volunteer subjects were moulaged with simulated burn injuries of 25%, 30% and 35% total body surface area (TBSA), respectively. Various healthcare workers were invited to use both the 3D Burn application as well as the conventional methods stated above to estimate the volunteer subjects’ burn percentages.

**Results:**

Collective relative estimations across the groups showed that when used, the Rule of Palm, Rule of Nines and the Lund and Browder chart all over-estimated burns area by an average of 10.6%, 19.7%, and 8.3% TBSA, respectively, while the 3D Burn application under-estimated burns by an average of 1.9%. There was a statistically significant difference between the 3D Burn application estimations versus all three other modalities (*p* < 0.05). Time of using the application was found to be significantly longer than traditional methods of estimation.

**Conclusions:**

The 3D Burn application, although slower, allowed more accurate TBSAB measurements when compared to conventional methods. The validation study has shown that the 3D Burn application is useful in improving the accuracy of TBSAB measurement. Further studies are warranted, and there are plans to repeat the above study in a different centre overseas as part of a multi-centre study, with a view of progressing to a prospective study that compares the accuracy of the 3D Burn application against conventional methods on actual burn patients.

## Background

Accurate total body surface area burned (TBSAB) estimation is a crucial aspect of early burn management. It helps guide resuscitation and is essential in the calculation of fluid requirements. Conventional methods of estimation can often lead to large discrepancies in burn percentage estimation. These inaccurate estimations can lead to a multitude of drawbacks such as unnecessary transfers to tertiary burn centres, as well as over or under resuscitation of burn patients. TBSAB is also an extremely useful predictor of mortality outcomes. It forms a critical aspect of mortality prediction models such as the Baux Index, which has been shown to be effective in predicting outcomes in 87% of patients aged 60 and above [1]. Studies have continually shown that there can be significant variability in the estimation of burn percentages depending on the assessment tool used and the assessors’ level of experience in managing burns [2]. Some studies have shown that despite the ability for physicians to accurately sketch out TBSAB, there was still significant over-estimation and inter-rater variability [3]. Over-estimations of up to 20% total body surface area (TBSA) have been noted in studies on the Rule of Palm [4, 5], and the Rule of Nines has been shown to be inaccurate in those with high body mass indices (BMI) [6].

We aim to compare a new method of burn estimation using a smart-phone application named 3D Burn Resuscitation (3D Burn) against conventional methods of estimation—Rule of Palm, Rule of Nines and the Lund and Browder chart. Conventional methods are two dimensional (2D) which can make them relatively inaccurate and laborious to complete.

In 2011, the free-to-use 3D Burn application was developed in Thailand. It was initially developed as a TBSAB measurement tool meant to be specific to the Asian population; quoting a difference in Asian and Caucasian physical structure, the latter of which most TBSAB measurement tools are based on. Although a few similar computerised burn estimators have been developed, to our knowledge, this is the only application which has employed the use of 3D body scanning technology on Asian-specific physique to develop the models within the application. Other computerised burn estimators have different methods of burn estimation, for example, the German developed “Rapid Burn Assessor” [7] uses everyday objects, such as a mobile phone or credit card as a reference to objectively determine the size of burn injuries. The “PLoS One cloud-based consultation and smartphone application” similarly allows users to “paint” estimation of burn injuries albeit with the help of photographs, but the subsequent TBSAB calculation is done in a sub-application which is based on the Lund and Browder chart [8].

We hope to validate the aforementioned 3D Burn application, in hopes of identifying a faster, more accurate method of estimating burn percentages. With the advent of mobile phone technology, this smart-phone application could particularly be used to great advantage in the early stages of burn management both in the emergency department and in the immediate first-responder setting.

## Methods

Three volunteer subjects were moulaged with simulated burn injuries of 25%, 30% and 35% TBSA, respectively; this was measured and marked out as accurately as possible based on their estimated body surface area (BSA) [9]. The MedCalc: BSA, BMI calculator [9] was used to determine the volunteers estimated TBSA. Based on the measurements obtained from the calculator, simulated burn injuries of 25%, 30% and 35% TBSA, respectively, were then marked out on the volunteers. Flexible transparent plastic sheets corresponding to 1% TBSA of the subjects were measured out in square centimetre and used to trace out the simulated burn injuries.

**Fig. 1:**
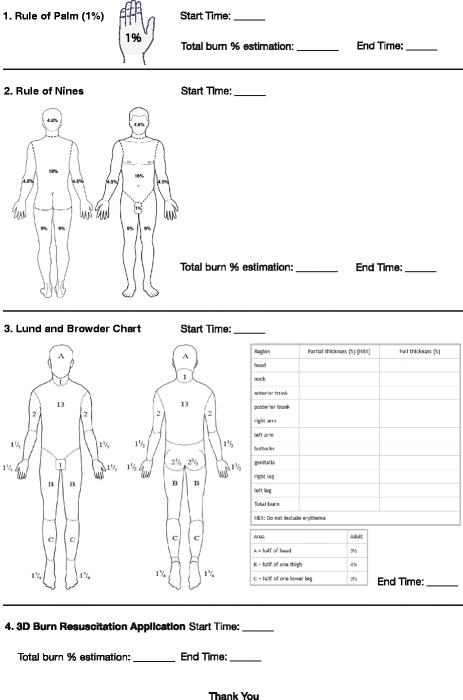
Study participant sheet

**Fig. 2:**
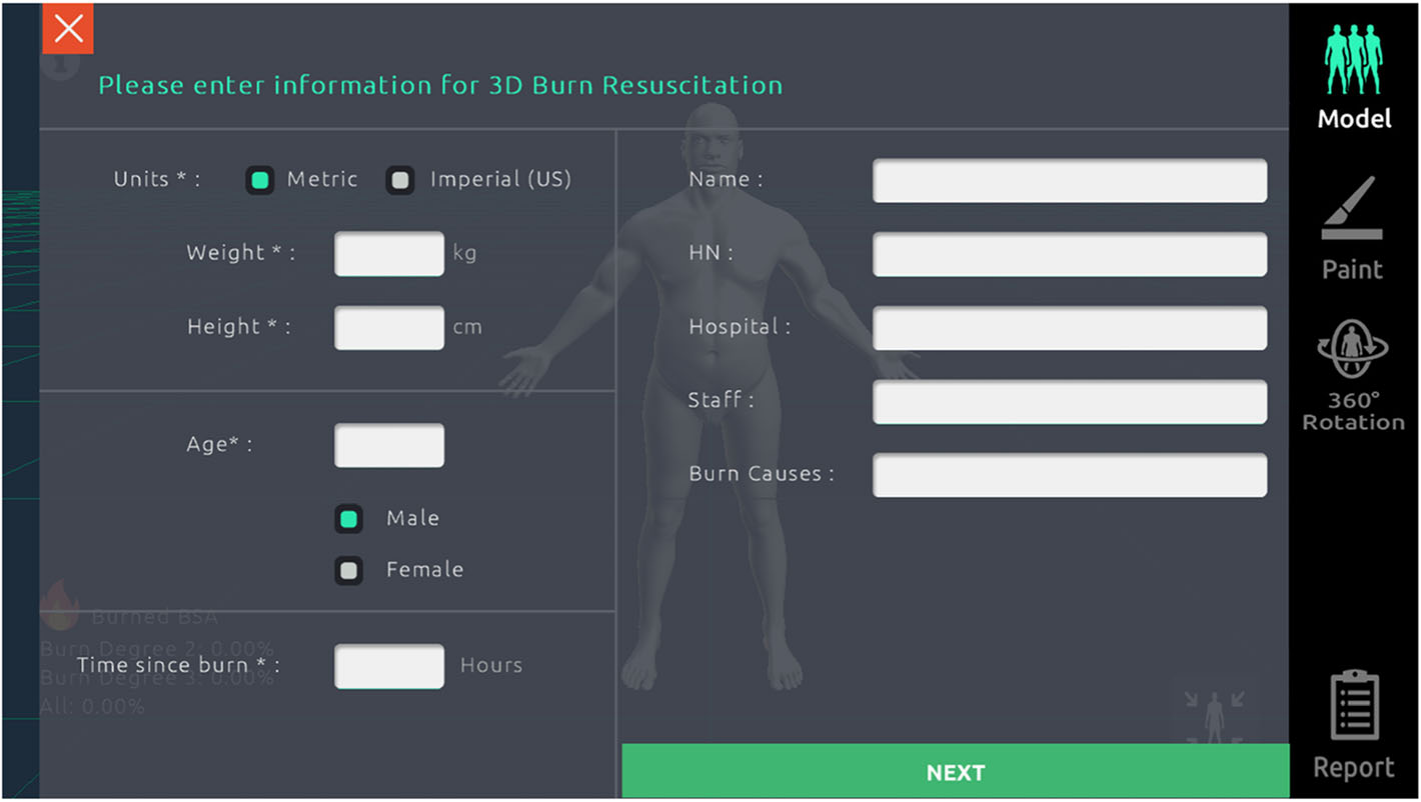
3D Burn Resuscitation application initial data screen

The study was conducted in conjunction with the 2017 Singapore Annual Burns Update, with participants from both local and international institutions. Various participants (Singapore Civil Service Defence Force—Paramedics/Firefighters, Singapore Armed Forces, Nurses and Physicians) were invited to use both the 3D Burn application as well as conventional methods of estimation: Rule of Palm, Rule of Nines and the Lund and Browder chart to estimate the volunteer subjects’ TBSAB (Figs. 1 and 2). Time to complete the estimation by each methods were recorded.

**Fig. 3:**
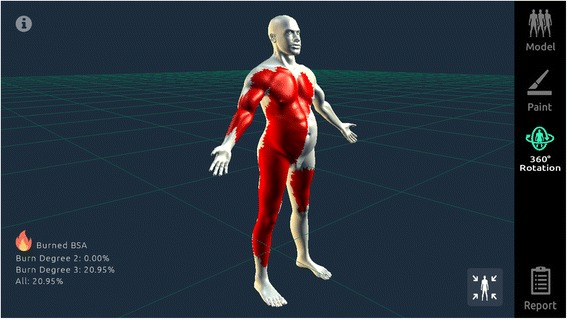
Burned areas are “painted” on the model in the 3D Burn Resuscitation application

The 3D Burn application allows users to “paint” burn injuries on the on-screen models—this then automatically computes TBSAB (Fig. 3).

A total of 82 participants took part in the validation study. Forty (49%) participants were male, and the remaining 42 (51%) were female. Sixty (73%) participants were between the ages of 25–40 years. Thirty-one (38%) participants had 1–5 years of experience in managing burns patients, 26 (32%) had 6–10 years of experience and 25 (30%) had beyond 10 years of experience. The participants could be broadly split into three categories—first-line responders (paramedics, firefighters and emergency response teams) formed the biggest group with 46% of responses. Nurses and physicians formed the next two groups with 34% and 20% of responses, respectively (Table 1).

**Table 1:** Participants composition in the validation study of the 3D Burn Resuscitation application

	Subject A (25% TBSA) *n*	Subject B (30% TBSA) *n*	Subject C (35% TBSA) *n*
Physicians	8	5	3
Nurses	17	4	7
First-line responders	18	13	7
Total	43	22	17

*TBSA* Total body surface area

None of the participants had prior exposure or training with the 3D Burn application. A 15-min power-point-based tutorial was carried out on the day of the study itself. Participants were required to download and install the application on their own smart-phones.

### Statistical analysis

As the three different subjects had differing burn percentages, the paired *t* test was used to test each individual pair of data against the 3D Burn application. For example, the paired *t* test was used three times to assess the mean time to complete estimation from subject A (25% TBSA)—Rule of Palm versus (vs) 3D Burn application, Rule of Nines vs 3D Burn application and Lund and Browder chart vs 3D Burn application. The above was repeated for subject B (30% TBSA) and subject C (35% TBSA).

## Results

The results from the above study were tabulated and broadly categorised into a few different aspects—mean TBSAB estimations, relative estimations to actual TBSAB and the mean time to completion of each assessment tool. In total, there are three different sets of data—one for each subject (25%, 30% and 35% TBSA, respectively) (Table 2).

**Table 2:** Summary of TBSAB estimation by 3D Burn application and the conventional methods

	Subject A (25% TBSA) (*n* = 43)		Subject B (30% TBSA) (*n* = 22)		Subject C (35% TBSA) (*n* = 17)	
	%TBSAB estimation Mean (SD)	Relative to actual % TBSAB	%TBSAB estimation Mean (SD)	Relative to actual % TBSAB	%TBSAB estimation Mean (SD)	Relative to actual % TBSAB
Rule of Palm	36.40 (6.39)	+ 11.41	38.86 (13.42)	+ 8.86	46.54 (14.29)	+ 11.54
Rule of Nines	39.36 (6.13)	+ 14.36	51.90 (12.11)	+ 21.90	57.98 (14.29)	+ 22.98
Lund and Browder chart	28.87 (4.75)	+ 3.87	44.09 (4.78)	+ 14.09	42.08 (2.87)	+ 7.08
3D Burn application	21.07 (4.96)	-3.93	30.03 (7.43)	+ 0.03	33.14 (11.90)	− 1.86

*TBSA* Total body surface area, *TBSAB* Total body surface area burned, *SD* Standard deviation, *3D Burn* 3D Burn Resuscitation

In order to negate the possibility of difference in time to completion due to differing burn wound sizes, the time to completion was also tested in a similar fashion (Table 3).

**Table 3:** Mean time to completion by 3D Burn application and the conventional methods

Mean time to completion (min)			
	Subject A (25% TBSA) (*n* = 43)	Subject B (30% TBSA) (*n* = 22)	Subject C (35% TBSA) (*n* = 17)
Rule of Palm	2.7	3.1	3.5
Rule of Nines	2.3	3.1	3.5
Lund and Browder chart	2.0	3.1	4.9
3D Burn application	4.5	4.4	6.4

*TBSA* Total body surface area and *3D Burn* 3D Burn Resuscitation

Table 4 showed that there was a statistically significant difference in both TBSAB measurements and time to completion between the 3D Burn application estimations vs all three other modalities (Rule of Palm, Rule of Nines and Lund and Browder chart) (*p* < 0.05 [Table 4]). There was also a statistically significant difference in time to completion- usage of the application took significantly longer time compared to traditional methods of estimation.

**Table 4:** Comparison between 3D Burn application and other conventional methods in TBSAB estimation and time to complete the estimation

	Subject A (25% TBSA) (*n*=43)				Subject B (30% TBSA) (*n*=22)				Subject C (35% TBSA) (*n*=17)			
	TBSAB Measurement		Time to Completion		TBSAB Measurement		Time to Completion		TBSAB Measurement		Time to Completion	
	Paired *t*-test *t* value	Mean difference (%)	Paired *t*-test *t* value	Mean difference (min)	Paired *t*-test *t* value	Mean difference (%)	Paired *t*-test *t* value	Mean difference (min)	Paired *t*-test *t* value	Mean difference (%)	Paired *t*-test *t* value	Mean difference (min)
Rule of Palm vs 3D Burn application	14.32^***^	15.34	-3.86^***^	-1.86	3.29^**^	8.82	-2.20^*^	-1.27	4.01^**^	13.41	-2.95^**^	-2.46
Rule of Nines vs 3D Burn application	19.54^***^	18.29	-4.48^***^	-2.26	8.69^***^	21.87	-2.10^*^	-1.27	6.96^***^	24.84	-4.67^***^	-2.87
Lund and Browder chart vs 3D Burn application	12.11^***^	7.80	-4.74^***^	-2.52	8.62^***^	14.05	-2.01	-1.34	3.21^**^	8.94	-2.12^*^	-1.53

^*^P<0.05, ^**^P<0.01 and ^***^P<0.001 indicate significant differences

*TBSA* Total body surface area, *TBSAB* Total body surface area burned, *3D Burn* 3D Burn Resuscitation

### Burn estimations relative to actual TBSAB

Collective relative estimations across the subjects (A, B and C) showed that when used, the Rule of Palm, Rule of Nines and Lund and Browder chart all over-estimated burns by an average of 10.6%, 19.7% and 8.3% TBSA, respectively. The 3D Burn application under-estimated burns area by an average of 1.9%.

## Discussion

Accurate measurement of TBSAB remains a crucial, but highly subjective area in the management of burn patients. Inaccurate measurement leads to a multitude of problems, particularly in the form of unnecessary transfers to tertiary burn centres and over or under resuscitation of burn patients. The ideal form of TBSAB measurement would be one that is not only 3D, but one that is entirely objective, with no subjective element. Astounding overestimations of greater than 100% have been identified in some emergency departments [7, 10–13]. These inaccuracies are largely due to human error, further highlighting the subjective element of the current methods of TBSAB measurement.

The data from our study has shown promising results. The 3D Burn application, although slower, allowed more accurate TBSAB measurements when compared to conventional methods. With the advent of technology and the push towards electronic data, the 3D burn application could be a step in the right direction to reducing the subjective element of TBSAB measurement.

We recognise that this study has its limitations. The burn models or templates in the 3D Burn application may not be an entirely accurate representation of the actual burn patient—this may ultimately affect TBSAB accuracy on the application itself. Additionally, the study was conducted on a relatively small population of healthcare and frontline workers and although all participants had prior exposure to management of burn patients, there were varying levels of experience and not all were adept or familiar with the intricacies of TBSAB measurement. Although the difference in results between the 3D Burn application and traditional methods of TBSAB measurement were statistically significant, and the clinical significance is yet to be determined.

Finally, the practical implications of using a smart-phone application for TBSAB could also be of concern. Many participants in the study found the application more challenging to use compared to the traditional pen and paper methods of measurement. Some participants also questioned patient confidentiality—although the application itself does not require pictures or patient identifiers to function. Perhaps, an encrypted dedicated smart device with a larger screen could be something that improves the user experience and assures patient confidentiality.

## Conclusion

The validation study has shown that the 3D Burn application could be useful in improving the accuracy of TBSAB measurement. There are plans to address some of the limitations and repeat the study in a different centre. In addition to extending the study across subjects of differing body weights, we would also like to place emphasis on participants with more experience in burn TBSAB measurement to allow for a more accurate assessment. Results will be collated and analysed as part of a multi-centre study, with a view of progressing to a prospective study that compares the accuracy of the 3D Burn application against conventional methods on actual burn patients.

## Abbreviations

BMI: Body mass index
BSA: Body surface area
SD: Standard deviation
TBSA: Total body surface area
TBSAB: Total body surface area burned
VS: Versus
